# miR-532-3p Inhibits Proliferation and Promotes Apoptosis of Lymphoma Cells by Targeting β-Catenin

**DOI:** 10.7150/jca.45684

**Published:** 2020-06-06

**Authors:** Yan Liu, Qiuying Li, Yongzhou Dai, Tinghui Jiang, Yongming Zhou

**Affiliations:** 1 Department of Oncology, Putuo Hospital Shanghai University of Traditional Chinese Medicine, Shanghai 20062, China; 2 Department of Hematology, Yueyang Hospital of Traditional Chinese and Western Medicine, Shanghai University of Traditional Chinese Medicine, Shanghai 200437, China

**Keywords:** lymphoma, miR-532-3p, β-catenin, proliferation, apoptosis

## Abstract

**Background:** Dysregulated expression of miR-532-3p has been observed in several types of cancer and plays a key role in tumor progression and metastasis. In this study, we analyzed the role and molecular mechanism of miR-532-3p in lymphoma progression.

**Methods:** The expression of miR-532-3p in lymphoma sample tissues was analyzed using the GEO database and in cell lines by quantitative reverse transcription (qRT)-PCR. The functions of miR-532-3p in lymphoma cell proliferation and apoptosis were analyzed by CCK-8 assay and Annexin V-FITC/propidium iodide staining, respectively. *In vivo*, the tumor weight and volume were measured. The target gene of miR-532-3p was predicted using miRanda software, and then luciferase, qRT-PCR, and western blot assays were performed to verify that β-catenin was the downstream target gene of miR-532-3p.

**Results:** miR-532-3p was decreased in lymphoma tissues and cell lines. *In vitro* and *in vivo* experiments showed that overexpression of miR-532-3p inhibited lymphoma cell proliferation and promoted apoptosis. Mechanistic studies demonstrated that β-catenin was a functional target gene of miR-532-3p. Furthermore, we found that overexpression of β-catenin reversed the tumor-suppression activities caused by overexpression of miR-532-3p in lymphoma proliferation and apoptosis.

**Conclusion:** This study demonstrates that miR-532-3p functions as a tumor inhibitor in lymphoma progression by targeting β-catenin, suggesting miR-532-3p/β-catenin as a new diagnosis marker or potential therapeutic target in lymphoma.

## Introduction

Lymphoma is a malignant hematologic tumor that can invade nearly all tissues and organs in the body 1. The main treatment methods for lymphoma are surgery, chemotherapy, radiation, immunotherapy, and autologous stem cell transplantation 2-5. With advances in comprehensive treatment over the last two decades, the prognosis of lymphoma has significantly improved. However, approximately 10% of lymphomas relapse or become refractory after first-line therapy 6, 7, which is the main cause of poor prognosis in lymphoma patients 8. The underlying mechanism of lymphoma progression remains unclear. Therefore, it is necessary to explore the mechanism of lymphoma and identify potential molecular targets to improve long-term prognosis.

MicroRNAs (miRNAs) are a group of non-protein-coding RNAs, approximately 22 nucleotides in length. They negatively regulate downstream gene expression by binding to the 3' untranslated regions (UTRs) of their target genes and repressing mRNA translation 9-11. An increasing number of studies have suggested that miRNAs play important roles in biological processes and are involved in many human diseases, particularly cancer 12. Numerous studies have demonstrated the important regulatory roles of miRNAs in tumor progression, acting as oncogenes or tumor suppressors by regulating gene expression 13-15. miR-532-3p is reportedly downregulated in several types of tumors and is considered as a tumor suppressor 16-18. One report showed that miR-532-3p could predict the effectiveness of rituximab in patients with chronic lymphocytic leukemia 19; another study found that miR-532-3p was expressed at low levels in patients with classical Hodgkin lymphoma compared to controls 20. However, the biological function and underlying mechanism of miR-532-3p in lymphoma are unclear.

In this study, we examined whether the expression of miR-532-3p was downregulated in lymphoma tissues and cell lines compared to normal lymphocytes. Next, the effects of miR-532-3p on lymphoma cell proliferation and apoptosis were analyzed. Our results revealed that the β-catenin gene is directly targeted by miR-532-3p. These results suggest that miR-532-3p induces lymphoma progression by targeting β-catenin.

## Materials and Methods

### Data source and miRNA expression analysis

The date for miRNA expression in lymphoma (50 lymphoma tissues and 6 normal lymphoid tissues, GSE31377) were obtained from the Gene Expression Omnibus (GEO, https://www.ncbi.nlm.nih.gov/geo/). All GEO data were analyzed by GEO2R or R.

### Cell culture

The Jurkat, HUT102, U937, Raji, and lymphocyte cell lines were purchased from ATCC (Manassas, VA, USA) and cultured in RPMI-1640 medium (SH30809.01b, Hyclone, Logan, UT, USA) containing 10% fetal bovine serum (16000-044, Grand Island, NY, USA) and 1% double antibiotic (penicillin-streptomycin) solution (P1400-100, Beijing, China) in an incubator at 37 °C with 5% CO_2_.

### Cell transfection

The miR-532-3p mimic (5'-CCUCCCACACCCAAGGCUUGCA-3') and miR-532-3p inhibitor (5'-UGCAAGCCUUGGGUGUGGGAGG-3') were purchased from Shanghai GenePharma Co, Ltd. (Shanghai, China). The cells (3 × 10^5^ cells/well) were seeded into 6-cm plates and cultured overnight before transfection. When the cell density reached 70-80%, the cells were transfected using Lipofectamine 2000 (Invitrogen, Carlsbad, CA, USA) according to the manufacturer's instructions.

### Cell proliferation

Cell proliferation was analyzed with a Cell Counting Kit-8 (CCK-8, SAB, CP002). Cells (3 × 10^3^ cells/well) were seeded into a 96-well plate with 100 µL medium and cultured in an incubator for 0, 24, 48, and 72 h, and then with 10 µL CCK-8 in an incubator for 1 h. The optical density (OD) of each well was measured at 450 nm, and a graph of the absorbance values was generated.

### Cell apoptosis analysis

Cell apoptosis was detected with an Annexin V-FITC/PI kit (C1062M, Beyotime, Shanghai, China) according to the manufacturer's instructions. Cells (5 or 10 × 10^4^ cells/well), including the cell suspension, were collected after transfection for 48 h and washed twice with cold PBS, followed by resuspension in 195 μL binding buffer. Next, the cells were incubated with 5 μL Annexin V-fluorescein isothiocyanate (FITC) for 15 min, followed by 10 μL propidium iodide for 5 min at 4 °C in the dark. Flow cytometry was performed to detect cell apoptosis, and data were analyzed with BD Accuri c6 software (BD Biosciences, Franklin Lakes, NJ, USA).

### Real-time PCR (RT-PCR) analysis

Following the manufacturer's protocol, total RNA from cells or mouse tumor tissues was extracted with TRIzol reagent (Invitrogen). cDNA was synthesized using a RevertAid First Strand cDNA Synthesis Kit (Thermo Fisher Scientific, Waltham, MA, USA). Real-time PCR was conducted using Maxima SYBR Green/ROX qPCR Master Mix under the following thermal cycling conditions: 1) 95 °C for 10 min, 2) 95 °C for 15 s and then 60 °C for 45 s for 40 cycles, 3) 95 °C for 15 s, 4) 60 °C for 1 min, 5) 95 °C for 15 s, 6) 60 °C for 15 s. The relative content of miRNA was analyzed using the instrument software (ABI Prism 7300 SDS Software, Foster City, CA, USA).

### Western blotting analysis

Cell lines and mouse tissues were lysed using a RIPA lysis buffer kit (JRDUN Biotechnology, Shanghai, China), and total protein was quantified with a BCA quantification kit (Thermo Fisher Scientific). Total proteins (20 μg) were separated by 10% or 15% sodium dodecyl sulfate-polyacrylamide gel electrophoresis, and the samples were transferred to a polyvinylidene difluoride membrane (EMD Millipore, Billerica, MA, USA).The primary antibodies c-myc (1:1000) (Ab39688, Abcam, Cambridge, UK), proliferating cell nuclear antigen (1:1000) (#13110, Cell Signaling Technology, Danvers, MA, USA), Survivin (1:1000) (#2808, Cell Signaling Technology), cleaved-caspase3 (1:1000) (Ab2302, Abcam), β-catenin (1:1000) (#8480), and GAPDH (1:2000) (#5174, Cell Signaling Technology) were diluted to the desired concentrations by blocking buffer and incubated with the membrane at room temperature for 2 h or 4 °C overnight. The membrane was washed with Tris-buffered saline containing 5% Tween 20 three times and incubated with goat anti-rabbit horseradish peroxidase-labeled secondary antibody (1:1000) (A0208, Beyotime) at room temperature (37 °C) for 2 h. Electrochemiluminescence was used to visualize the protein bands, which were scanned with a Tanon-5200 (Tanon Science & Technology, Shanghai, China). Protein bands were analyzed with ImageJ 5.1 software (NIH, Bethesda, MD, USA), and the western blotting results were evaluated by semi-quantitative analysis. The relative protein expression means that the target protein density normalized to GAPDH, the internal reference protein.

### Luciferase assay

To predict the direct targets of miRNA-532-3p, PITA, miRmap, miRanda software (http://www.microrna.org//) were used. To construct the luciferase plasmid, β-catenin-3'UTR, and mutated β-catenin-3' UTR were inserted into the pGL3-Promoter vector (Invitrogen) to construct Wt-β-catenin-3'UTR or Mut-β-catenin-3'UTR. U937 cells (5 × 10^5^/well) were seeded into 6-well plates and cultured in an incubator for 24 h, after which the cells were co-transfected with the luciferase reporter vectors Wt-β-catenin 3'UTR or Mut-β-catenin 3'UTR and 5 µL miRNA-532-3p mimics or negative control (NC) mimics using Lipofectamine 2000 (Invitrogen). At 48 h after transfection, firefly and Renilla luciferase activities were measured using the Dual Luciferase Kit (Promega, Madison, WI, USA) according to the manufacturer's protocol.

### Tumor growth in nude mice

Female BALB/c nude mice (4-6-weeks old, weighting 18-20 g) were obtained from the Shanghai Experimental Animal Center, Chinese Academy of Sciences. All mice were housed in a specific pathogen-free laboratory. Animal experiments were performed with the approval of the Animal Ethics committee of Tongji University, Shanghai. Twelve mice were randomly divided into the NC and mimic groups, with six mice in each group, and subcutaneously injected with 100 μL (5 × 10^7^ cells/mL) U937 cells in the right flank. After tumorigenesis, mice were administered NC or miRNA-532-3p mimics via tail vein injection for 3 weeks (0.5 OD/day). Tumor length and width were measured every 3 days, until the end of the experiment. The tumor volume was calculated as follows: tumor volume = (length × width^2^)/2. All mice were sacrificed 33 days after the first injection, the tumors were removed and weighed, and tumor samples were stained with hematoxylin and eosin.

### Plasmid overexpression

The CDS (coding sequence) of β-catenin was searched using the Gene database in NCBI (https://www.ncbi.nlm.nih.gov/), and the primers were designed. The CDS was then amplified from complementary DNAs of U937 cells by PCR using the following primers: Forward: 5'-CCCAAGCTTATGGCTACTCAAGCTGATTTGATG-3'; Reverse: 5'-CGGAATTCTTACAGGTCAGTATCAAACCAGGC-3' (Hind III, EcoR I sites were introduced). The amplification product and pcDNA3.1(+) vector (Addgene, Rockville, MD) were digested using Hind III and EcoR I endonucleases, respectively. Then the amplification product was inserted into pcDNA3.1(+) catalyzed by T4 DNA ligase (Thermo Fisher). The β-catenin overexpression plasmid sequences were verified by DNA sequencing.

### Statistical analysis

All experiments were repeated three times independently. All data were expressed as mean ± SD and analyzed with GraphPad Prism 7.0 software (GraphPad, Inc., La Jolla, CA, USA). Differences between two groups or more than two groups were analyzed by Student's *t*-test or analysis of variance. *P* < 0.05 was considered to indicate statistically significant results.

## Results

### Expression of miR-532-3p is downregulated in lymphoma tissues and cell lines

**Figure 1:**
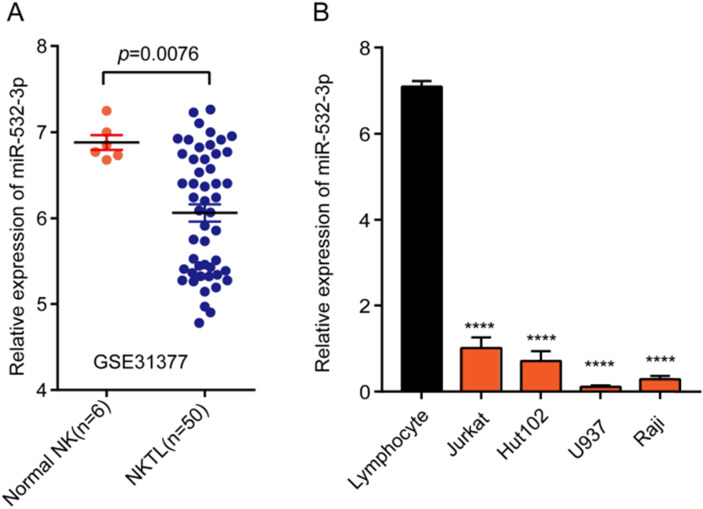
** Expression of miR-532-3p in both lymphoma tissues and cell lines. (A)** The expression level of miRNA-532-3p in lymphoma samples (n = 50) was lower than that in normal lymphoid tissues (n = 6). **(B)** miR-532-3p expression in lymphoma cell lines was lower as compared to lymphocytes. ****P < 0.0001.

To explore the promoting or suppressive effects of miR-532-3p in lymphoma, we analyzed microarray data from GEO datasets. Our results showed that miR-532-3p expression was significantly downregulated in lymphoma tissues (Figure 1A). To further evaluate the miR-532-3p expression in lymphoma cell lines, we performed RT-PCR. As shown in Figure 1B, the expression of miR-532-3p in lymphoma cell lines (Jurkat, Hut102, U937, Raji) was downregulated compared to normal lymphocytes.

### miR-532-3p suppresses cell proliferation in lymphoma

**Figure 2:**
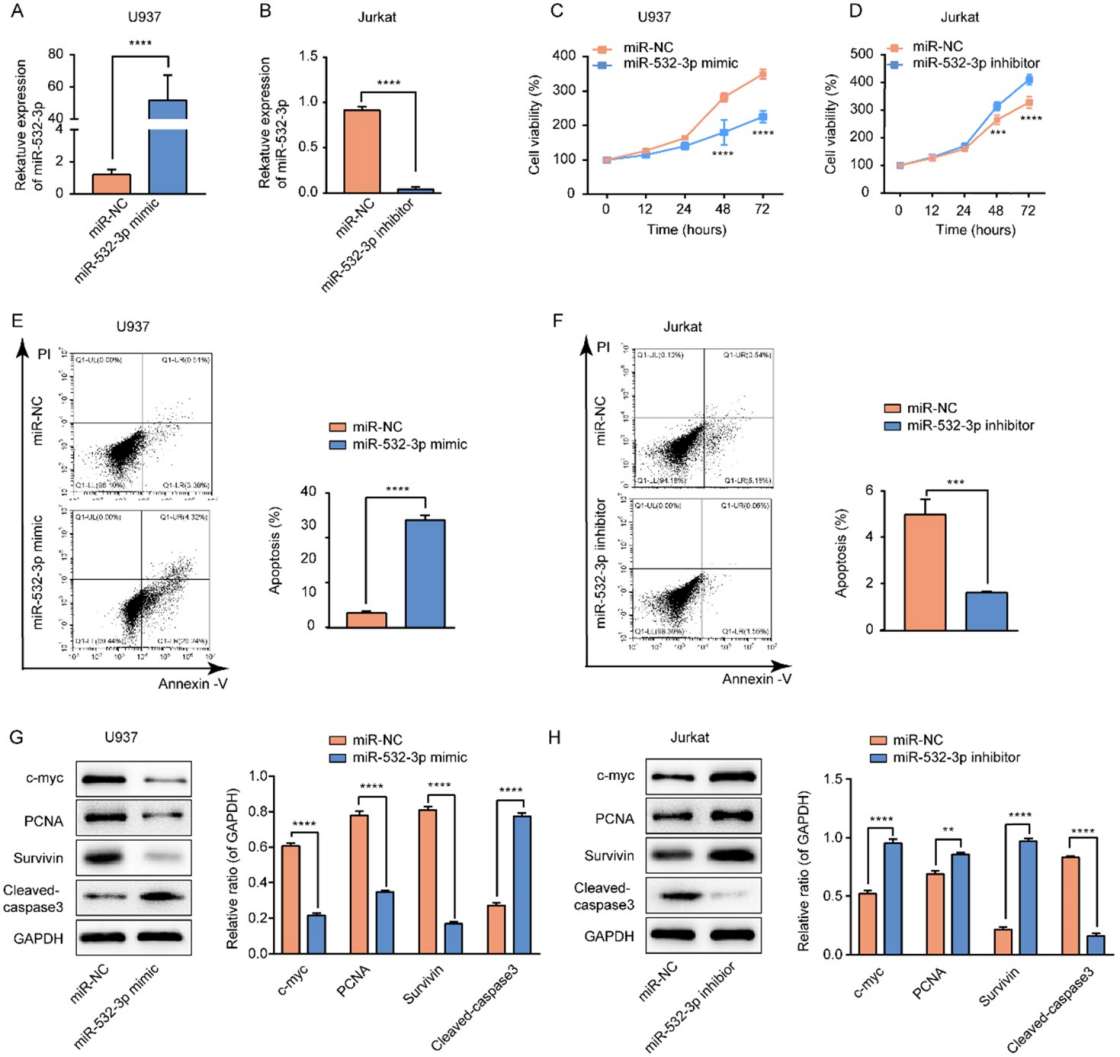
** Effects of miR-532-3p on proliferation and apoptosis in lymphoma cells. (A, B)** U937 and Jurkat cells were transfected with miR-532-3p mimic/NC and a miR-532-3p inhibitor/NC, respectively; miR-532-3p expression was analyzed by RT-PCR. **(C, D)** Cell viability of U937 and Jurkat cells transfected miR-532-3p mimic, or miR-532-3p inhibitor, were detected by CCK-8 assay. **(E, F)** U937 and Jurkat cells transfected with miR-123-3p mimics or miR-532-3p inhibitor were stained by Annexin V/PI and analyzed by flow cytometry after 48 hours following transfection. **(G, H)** The level of c-myc, PCNA, Survivin, and cleaved-caspase3 were evaluated by western blotting. Data are expressed as the mean ± SD of triplicate experiments. *P < 0.05, **P < 0.01, ***P < 0.001, ****P < 0.0001, ns. non-significant.

We transfected the miR-532-3p mimic/NC and miR-532-3p inhibitor/NC into U937 and Jurkat cells, respectively. The expression of miR-532-3p was increased in miR-532-3p mimic-transfected U937 cells (Figure 2A) and decreased in miR-532-3p inhibitor-transfected Jurkat cells (Figure 2B). The CCK-8 assay was performed to determine the function of miR-532-3p in lymphoma cell proliferation. We observed that miR-532-3p overexpression inhibited U937 cell proliferation (Figure 2C), whereas knockdown of miR-532-3p in Jurkat cells promoted cell growth (Figure 2D). In accordance with the previous results, expression levels of the cell proliferation-related proteins c-myc, proliferating cell nuclear antigen, and Survivin were decreased in the miR-532-3p mimic group (Figure 2G) and increased in the miR-532-3p inhibitor group compared to the NC group (Figure 2H). These results indicate that upregulated miR-532-3p inhibits lymphoma cell proliferation.

### miR-532-3p induces cell apoptosis in lymphoma

To further investigate whether miR-532-3p expression affects the apoptosis of lymphoma cells, we performed a flow cytometry assay. The results revealed that upregulated miR-532-3p significantly induced lymphoma cell apoptosis compared to the control group in U937 cells (Figure 2E), whereas decreased expression of miR-532-3p inhibited apoptosis compared to the control group in Jurkat cells (Figure 2F). We found that expression of the cell apoptosis-related protein cleaved-caspase3 was upregulated in the miR-532-3p mimic group (Figure 2G) but decreased in the miR-532-3p inhibitor group (Figure 2H). These results indicate that miR-532-3p overexpression induces apoptosis in lymphoma cells.

### miR-532-3p prevents lymphoma growth *in vivo*

**Figure 3:**
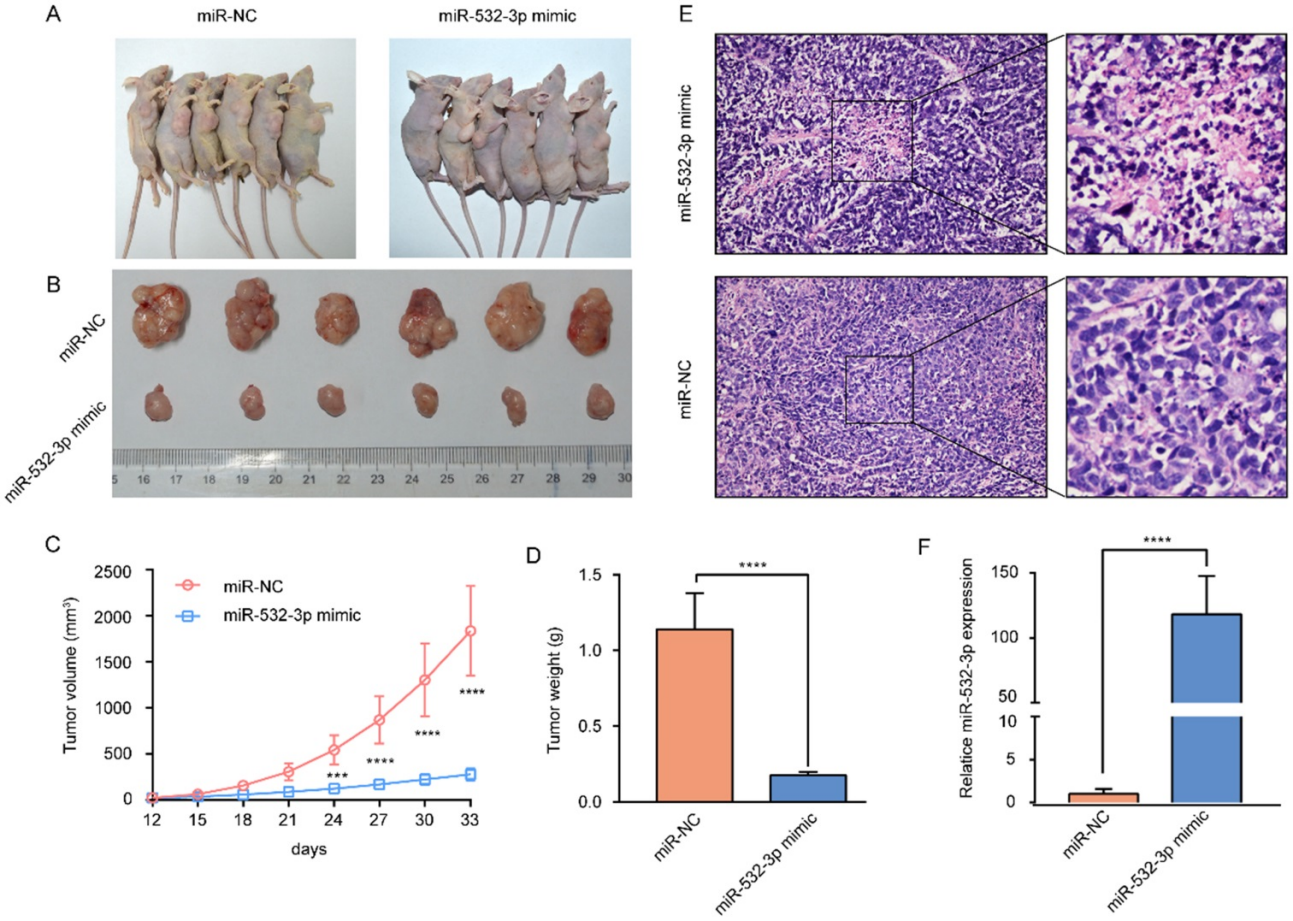
** miR-532-3p mimic inhibited lymphoma tumor growth *in vivo*. (A)** Image of subcutaneous tumor in mice of miR-NC and miR-532-3p mimic group. **(B-D)** Tumor weight and volume were measured, and the data are shown as the mean ± SD. **(E)** HE staining of tumor in nude mice. **(F)** RT-PCR analysis to compare miR-532-3p expression between miR-532-3p mimic tumor and control isolated from mice. ***P < 0.001, ****P < 0.0001.

To further investigate the effect of miR-532-3p on lymphoma growth *in vivo*, U937 cells transfected with the miR-532-3p mimic and control U937 cells were subcutaneously injected into nude mice. The results showed that tumors containing the miR-532-3p mimic were smaller and had a lighter weight than those in the NC group (Figure 3A-D). Hematoxylin and eosin staining of murine tumor tissues revealed that miR-532-3p mimic-treated tumors have a more disordered histopathological structure and morphology than the tumor samples from the NC group (Figure 3E). Additionally, RT-PCR was performed to confirm the expression of miR-532-3p in tumor tissue in each group, which showed miR-532-3p overexpression in the mimic group (Figure 3F). These results demonstrated that miR-532-3p overexpression suppressed lymphoma tumor growth *in vivo*.

### β-Catenin is a direct target of miR-532-3p in lymphoma

**Figure 4:**
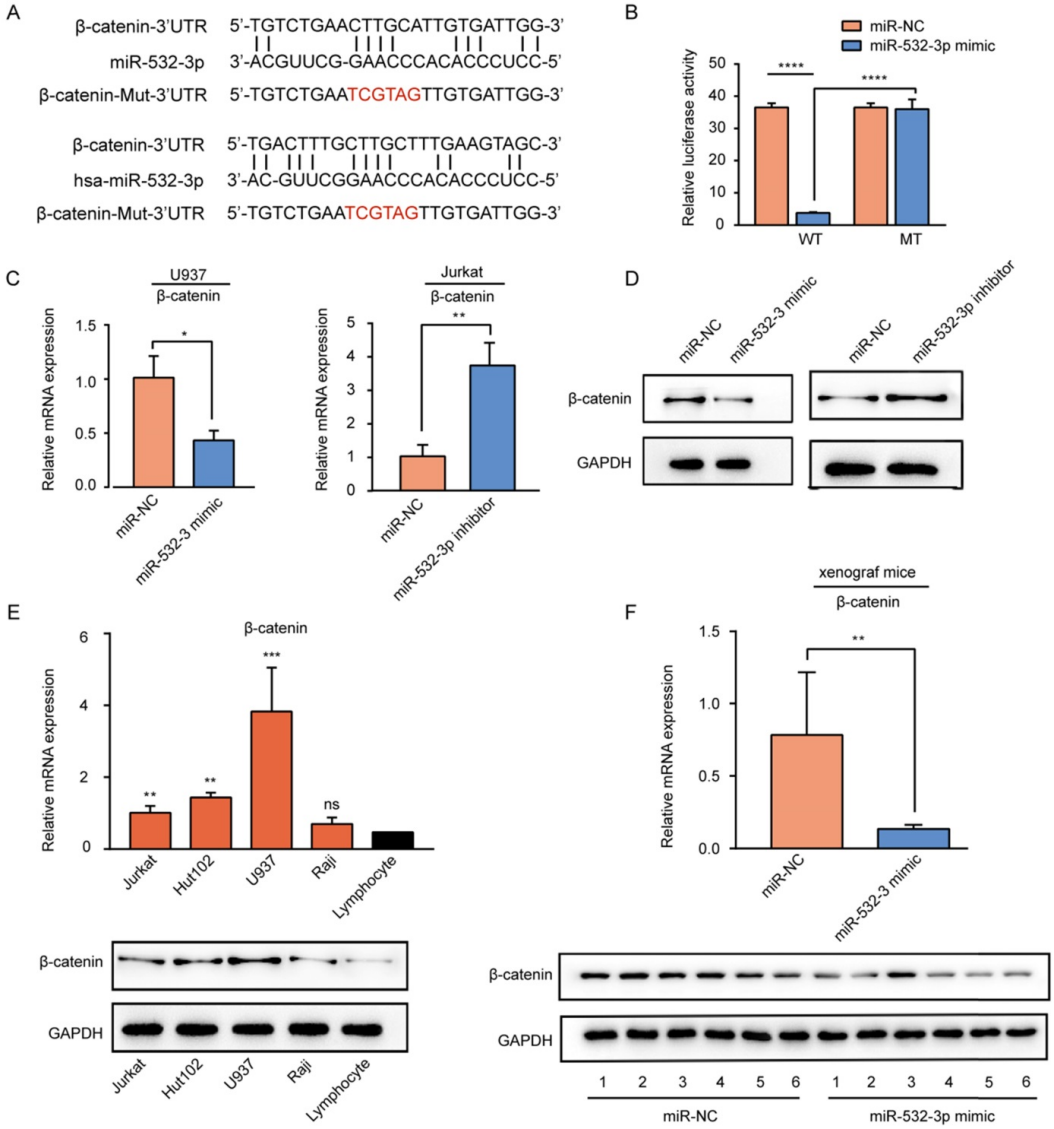
** β-catenin is the target gene of miR-532-3p. (A)** Binding sites between β-catenin and miR-532-3p were identified by miRanda. **(B)** Luciferase activity of the Wt-β-catenin 3'UTR or Mut-β-catenin 3'UTR after the transfection with miR-532-3p mimic or NC. **(C, D)** The mRNA and protein expression of β-catenin in U937 cells transfected miR-532-3p mimic or in Jurkat cells transfected miR-532-3p inhibitor. **(E)** The mRNA and protein expression of β-catenin in lymphoma cell lines (Jurkat, Hut102, U937, Raji) and lymphocyte. **(F)** RT-PCR and WB analysis to compare β-catenin expression between miR-532-3p mimic tumors and control tumors isolated from xenograft mice. *P < 0.05, **P < 0.01, ***P < 0.001, ns. non-significant.

To predict the downstream target gene of miR-532-3p, miRanda software was used. The predicted miR-532-3p binding site in the 3'UTR of β-catenin was mutated (Fig 4A). To further verify whether β-catenin was a target gene of mir-532-3p, we used luciferase reporter experiments. The results (Figure 4B) of the dual-luciferase reporter assay revealed that luciferase activity was decreased when the Wt-β-catenin group was transferred with the miR-532-3p mimic (****P < 0.0001), whereas in the Mut-β-catenin group, the luciferase activity was not changed in the miR-532-3p mimic group compared to that in the miR-NC group. As expected, RT-PCR (Figure 4C) and western blot (Figure 4D) analyses showed that the mRNA and protein expression levels of β-catenin were lower in the miR-532-3p mimic group than in the NC group in U937 cells, but higher in the miR-532-3p inhibitor group than in the NC group in Jurkat cells. We also found that the mRNA and protein expressions of β-catenin were upregulated compared to those in lymphocytes (Figure 4E). Additionally, the mRNA and protein expression levels of β-catenin were significantly lower in miR-532-3p mimicked tumors of xenograft mice than in the NC group (Figure 4F). These results indicate that β-catenin is a direct target gene of miR-532-3p.

### β-Catenin overexpression reverses the effects of miR-532-3p mimic in lymphoma cells

**Figure 5:**
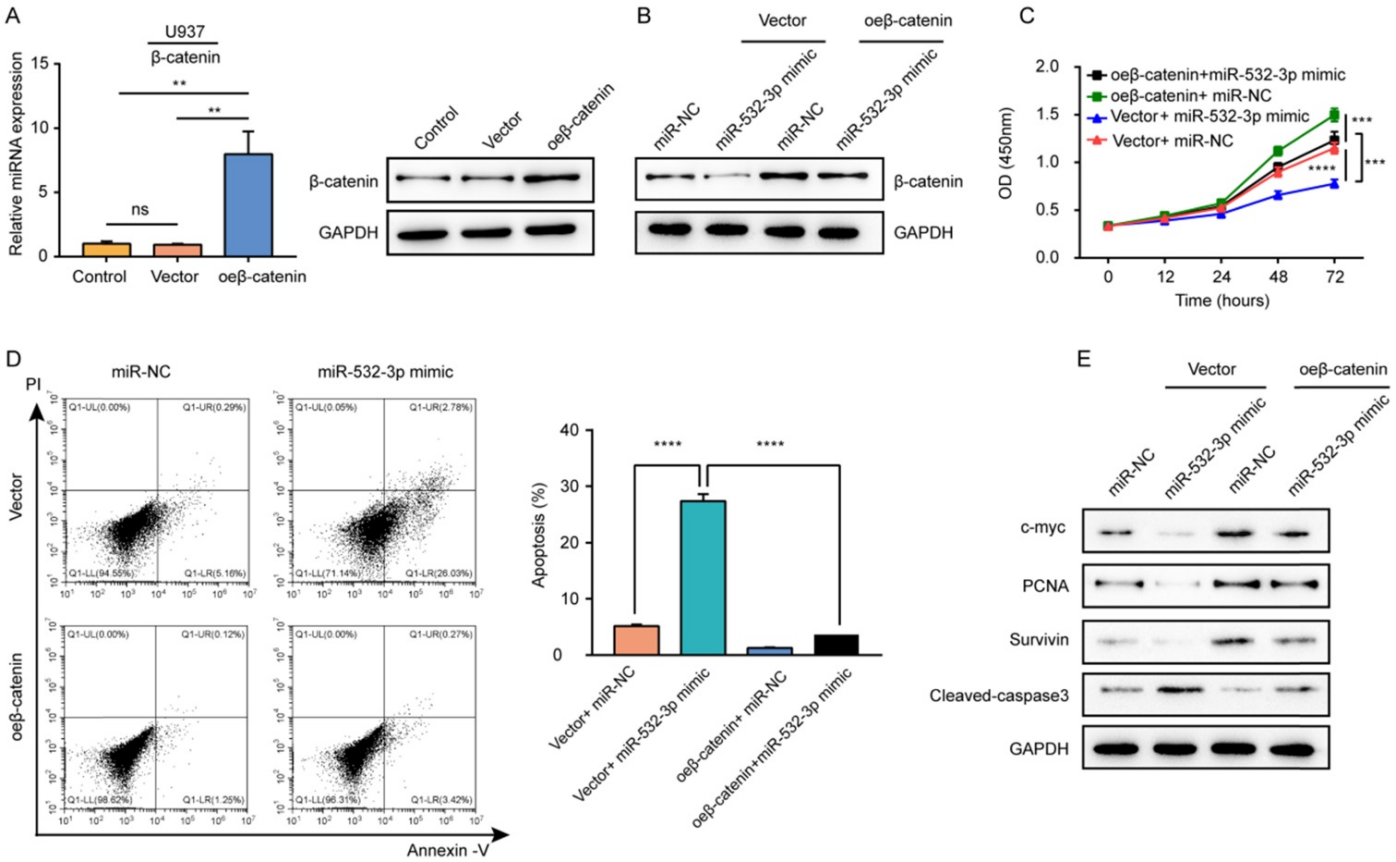
** β-catenin overexpression reversed the inhibitory effects of miR-532-3p on the proliferation, apoptosis of lymphoma cells. (A)** RT-PCR and Western blot analyzed the β-catenin expression in U937 cells transfected with control, vector, or oe-β-catenin. **(B)** The protein expression of β-catenin, **(C)** cell viability, **(D)** apoptosis cells, and **(E)** protein expression of c-myc, PCNA, Survivin, Cleaved-caspase 3 were measured in the four groups (Vector +miR-NC, Vector+miR-532-3p mimic, oeβ-catenin +miR-NC, oeβ-catenin+miR-532-3p mimic). **P < 0.01, ***P < 0.001, ****P < 0.0001.

To explore whether β-catenin mediates the biological functions of miR-532-3p in lymphoma, we carried out rescue experiments. First, the mRNA and protein expressions of β-catenin were detected in U937 cells transfected with the β-catenin overexpression vector to confirm the overexpression efficiency (Figure 5A). Rescue experiments showed that compared to its level in cells transfected with the miR-532-3p mimic, the protein expression of β-catenin increased after co-transfection with the miR-532-3p mimic overexpressing β-catenin (Figure 5B). The cell viability inhibition (Figure 5C) and apoptosis-inducing effects (Figure 5D) associated with the miR-532-3p mimic were rescued by overexpression of β-catenin. Furthermore, western blotting revealed that the expression of cleaved caspase 3 was decreased, whereas the levels of c-myc, proliferating cell nuclear antigen, and Survivin were upregulated in lymphoma cells after co-transfection with the miR-532-3p mimic and vector for β-catenin overexpression compared to those transfected with the miR-532-3p mimic (Figure 5E). Thus, β-catenin overexpression reversed the function of miR-532-3p that inhibited proliferation and promoted apoptosis in lymphoma cells.

## Discussion

Lymphoma is among the most common types of blood tumors and originates from the lymphatic hematopoietic system 1. The major causes of the poor prognosis of patients with lymphoma are the disease's recurrence and refractory state 7, 21. Although treatment methods for lymphoma have progressed, therapeutic options for relapsing and refractory lymphoma remain limited as the underlying molecular mechanism remains unclear 22, 23. Therefore, a better understanding of the molecular mechanism of lymphoma proliferation can lead to the development of targeted therapies.

Recent studies have reported that the expression of miR-532-3p, which suppresses cancer growth, was downregulated in several types of tumors, such as renal carcinoma, hepatocellular carcinoma, colorectal cancer 16-18, 24. However, the specific functions of miR-532-3p in lymphoma remain unclear. In this study, we have firstly shown that miR-532-3p was downregulated in lymphoma tissues and cell lines. Moreover, functional analysis of miR-532-3p in lymphoma cell lines and a nude mouse model showed that a miR-532-3p mimic suppressed tumor growth and progression and induced apoptosis, acting as an inhibitory oncomiR. Our results are consistent with those of previous studies showing that miR-532-3p inhibited tumor cell proliferation in lung cancer and gastric cancer 25, 26. Therefore, these findings demonstrated the effectiveness of using miR-532-3p as a molecular marker of lymphoma.

One question persists; how does miRNA-532-3p play a tumor-suppressive role in lymphoma? Recent reports have identified many targets that are regulated by miR-532-3p. Among which, PTPRT, FOXP3, and CCR7 that function in proliferation and metastasis, have been widely studied 17, 18, 25. In this study, β-catenin was found to be the target gene of miR-532-3p by miRanda software. Wnt/β-catenin is the most critical signaling pathway in some tumors. Previous studies demonstrated that overexpression of β-catenin promoted cancer phenotypes, including cell growth, metastasis, and invasion, and led to poor outcomes in many types of cancer 27-30. Low-expression of β-catenin has been reported to suppress cancer cell proliferation and induce apoptosis 31, 32. Taken together, these observations reveal that β-catenin acts as a promoter in tumor proliferation and metastasis, which is opposite to the inhibitory effect of miR-532-3p. In the present study, our data indicated that β-catenin expression was downregulated in the miR-532-3p mimic group, both *in vivo* and *in vitro*. Additionally, the protein and mRNA expression levels of β-catenin were increased in lymphoma cell lines. These results indicated that β-catenin is miR-532-3p's direct target gene. Simultaneously, considering that β-catenin promoted tumor progression in many types of cancer, it might be a significant mediator of the tumor-inhibitory functions of miR-532-3p. Moreover, overexpression of β-catenin reversed the effects of the miR-532-3p mimic in suppressing proliferation and inducing apoptosis in lymphoma cells, suggesting that miR-532-3p regulates these phenotypes by targeting β-catenin. Therefore, these results contribute to the hypothesis that miR-532-3p targeted β-catenin to suppress lymphoma progression. Since the exact mechanisms of β-catenin in lymphoma are still unclear, we need to investigate further the mechanism of β-catenin promoting the progression of lymphoma.

In conclusion, we demonstrated that miR-532-3p expression was downregulated in lymphoma cell lines and tissues, which promoted lymphoma progression. Moreover, we identified β-catenin as a downstream target gene of miR-532-3p and mediation of the biological function of miR-532-3p in lymphoma. Taken together, the miR-532-3p/β-catenin axis might be a new molecular mechanism in the progression of lymphoma, and miR-532-3p may be a useful prognostic marker and potential therapeutic target for treating lymphoma.

## Abbreviations

GEO: Gene Expression Omnibus
miRNA: microRNA
qRT-PCR: quantitative real-time polymerase chain reaction
UTR: untranslated region
